# CT diagnosis of small bowel obstruction caused by internal hernia from persistent attachment of a Meckel’s diverticulum to the umbilicus by the obliterated omphalomesenteric duct

**DOI:** 10.1259/bjrcr.20150131

**Published:** 2015-12-01

**Authors:** Davide Bellini, Marco Maria Maceroni, Giuseppe Cavallaro, Domenico De Santis, Olga Iorio, Marco Rengo, Gianfranco Silecchia, Andrea Laghi

**Affiliations:** ^1^ Department of Radiological Sciences, Oncology and Pathology, Sapienza–University of Rome, Latina, Italy; ^2^ Department of Medico-Surgical Sciences and Biotechnologies, Sapienza University, Rome, Italy

## Abstract

We report a case of small bowel obstruction (SBO) caused by internal hernia from persistent attachment of a Meckel’s diverticulum (MD) to the umbilicus by the obliterated omphalomesenteric duct that was diagnosed by multidetector CT and confirmed during laparoscopic surgery. Although clinical, pathological and radiological features of MD and its complications are well known, the diagnosis of MD is difficult to establish preoperatively. CT findings that allow the diagnosis of this very unusual cause of SBO are presented here with laparoscopic surgery correlation.

## Summary

We report a case of small bowel obstruction (SBO) caused by internal hernia from persistent attachment of a Meckel’s diverticulum (MD) to the umbilicus by the obliterated omphalomesenteric duct that was diagnosed by multidetector CT (MDCT) and confirmed during laparoscopic surgery. Although clinical, pathological and radiological features of MD and its complications are well known, the diagnosis of MD is difficult to establish preoperatively. CT findings that allow the diagnosis of this very unusual cause of SBO are presented here with laparoscopic surgery correlation.

## Background

MD is the most common congenital anomaly of the gastrointestinal tract, occurring in 2–3% of the population.^1^ It is a true diverticulum composed of all layers of the intestinal wall and resulting from an incomplete atrophy of the omphalomesenteric duct.^2^ It was first described by Fabricus Hildamus in 1598, and was defined embryologically by the German anatomist Johann Friedrich Meckel in 1809.^3^

About 25% of Meckel’s diverticula become symptomatic.^4^ Symptoms are secondary to complications, with the most common ones being haemorrhage, diverticulitis and intestinal obstruction from intussusception, torsion or volvulus. Internal hernia from persistent attachment of the MD to the umbilicus by the obliterated omphalomesenteric duct is a peculiar cause of bowel obstruction.

## Case report

**Figure 1.**
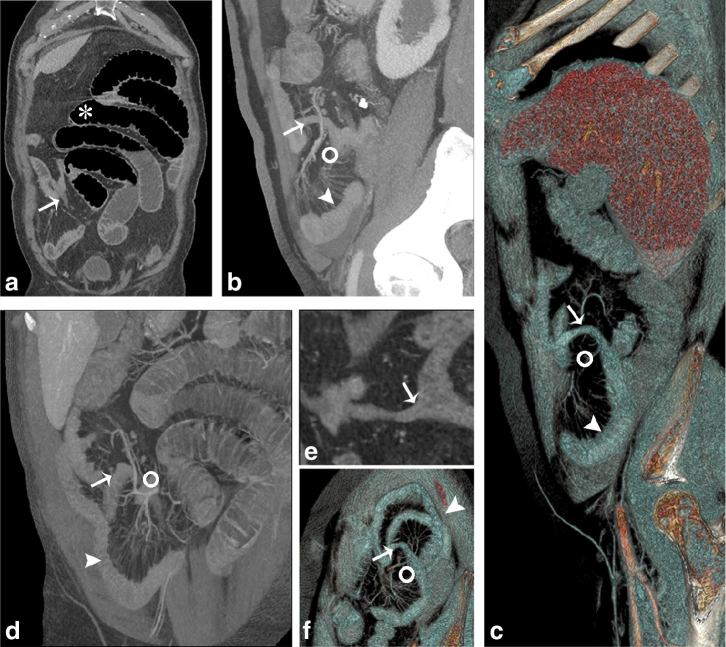
(a) Coronal reconstruction; (b) sagittal MIP reconstruction; (c) volume rendering parasagittal view; (d) oblique MIP reconstruction; (e) curved reconstruction; (f) volume rendering paracoronal view. Coronal reconstruction (a) of contrast-enhanced CT scan shows diffuse small intestine dilatation (asterisk) with multiple air–fluid levels. The transition point between the distended and the collapsed bowel loops is located at the level of a fibrous connection between the MD (arrow) and the anterior abdominal wall. A cluster of collapsed bowel loops (arrowheads; b, c, d and f) herniated throughout this orifice (circles in b, c, d and f) and configuring an internal hernia is also clearly visible. Figure (e) represents a curved reconstruction that shows the origin the MD from the ileal antimesenteric border (arrow). MD, Meckel’s diverticulum; MIP, maximum intensity projection.

A 70-year-old male was admitted to our hospital complaining of abdominal pain, nausea and vomiting for 3 days. The pain was accompanied by a loss of appetite, with no gas or faecal discharge within the previous 24 h. His past medical history revealed appendectomy. His vital signs were within the normal range and physical examination revealed distention of the abdomen with right lower quadrant tenderness. Laboratory tests were normal with the exception of a mild leukocytosis (11,800 cells mm^3^). Abdominal plain film showed findings of mechanical bowel obstruction and an abdominal CT was requested. The CT scan showed mildly distended ileal loops with fluid levels, clasped at the level of a fibrous connection between the MD and the abdominal wall. A cluster of collapsed bowel loops, approximately 50 cm, was herniated throughout this orifice, configuring an internal hernia (Figure 1). No signs of infarction were observed on the CT scan (Supplementary Video A).

**Figure 2.**
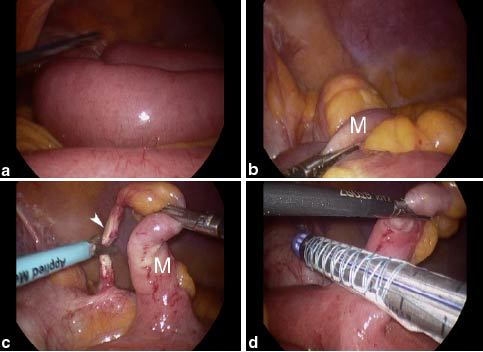
Laparoscopic exploration by 30°, 5-mm laparoscope revealed distension of the small bowel (a). In (b) laparoscopic view of focus of the bowel obstruction is reported. The ileum was incarcerated inside the hernia orifice between the MD (M) and the abdominal wall. (c) Serosal surface of a  5.6 cm MD (M) located on the antimesenteric side of the ileum was revealed. The fibrous band (arrowhead) is also clearly visible. In (c) and (d) the surgical procedure is reported: the fibrous band was released, the incarcerated bowel was normalized and the MD was sectioned by a laparoscopic linear stapler. MD, Meckel’s diverticulum.

The patient was immediately referred for surgery. Laparoscopic exploration revealed distension of the small bowel, confirming the presence of an internal hernia throughout an orifice caused by adhesion of the MD to the abdominal wall. The fibrous band was released, the incarcerated bowel was normalized and the MD was sectioned by a laparoscopic linear stapler (Figure 2). The entire small bowel was then carefully inspected and placed in its correct position. The patient was discharged after 5 days without postoperative complications.

## Discussion

MD is the most common congenital anomaly of the gastrointestinal tract. It is located within 100 cm of the ileocecal valve, at the antimesenteric border of the terminal ileum and accounts for 90% of all omphalomesenteric duct anomalies.^5-7^

The mnemonic “rule of 2s” is usually used to refer to this condition: it occurs in 2% of the population; it is often about 2 inches (5  cm) in length; it is found within 2 feet (60–100 cm) of the ileocecal valve; it is twice more common in males and the patients are often <2 years old at presentation.^8^

It is generally discovered incidentally during radiological or surgical procedures. Morphological features such as length and base diameter are considered as predisposing factors associated with increased complication rates: long and narrow-base diverticula are associated with an increased risk of bowel obstruction and inflammation; short and large-base ones are prone to foreign body entrapment.

SBO is the most typical clinical presentation in the adult population, occurring in almost 36% of patients.^7^ Rutherford and Akers^9^ described five principal mechanisms of SBO sustained by MD: volvulus around the vitelloumbilical or mesodiverticular cord; intussusception (free MD as the leading point); bulge in inguinal hernia (Littre’s hernia); inflammation and fibrous band formation with adhesions; a cord or fibrous band connecting the tip of the diverticulum and the base of the mesentery.

In our patient, the abdominal CT scan showed mildly distended distal ileal loops with fluid levels, clasped at its base by a loop-like structure, the MD, located 50 cm proximal to the end of the ileocecal valve; the suspicion of SBO caused by an internal hernia through a fibrous band formed by adhesion of the tip of the MD to the abdominal wall was sustained (Figure 1) and confirmed during surgery.

Internal hernia accounts for 4.1% of SBOs.^10^ It is a common surgical emergency that requires early diagnosis and prompt treatment to avoid the high mortality rate associated with this condition (approximately 50%).^11,12^

Because clinical diagnosis of internal hernias is difficult (less than 10% of symptomatic Meckel’s diverticula are diagnosed preoperatively^7^), imaging studies may play an important role if accurate and reliable CT findings can be obtained. However, owing to the lack of extensive evaluation of CT findings in the radiological literature, the diagnosis of internal hernia with CT scan is still a challenge for the radiologist. The main CT finding of internal hernias include evidence of SBO; configuration of the obstructed loop, mesenteric changes and enhancement patterns of the bowel wall should be investigated accurately.

In conclusion, it is well known that CT is a valuable imaging modality for the detection and characterization of SBO. Sometimes is difficult to identify a trigger point and an MD as the trigger point^13^ is an uncommon cause to be taken into account.

## Learning points

A MD, despite its rarity, can be the leading point of an internal hernia associated with SBO. This condition should be taken into account and investigated because an early diagnosis and prompt surgery are crucial to prevent the sudden deterioration of patient’s vital signs. Performing MDCT with the appropriate technique and reporting of the results by a radiologist skilled in abdominal imaging is the best way to make the diagnosis.

## Consent

Written informed consent was obtained from the patient for publication of this case report, including accompanying images.
